# Development of Capsaicin-Containing Analgesic Silicone-Based Transdermal Patches

**DOI:** 10.3390/ph15101279

**Published:** 2022-10-18

**Authors:** Szabolcs László, István Z. Bátai, Szilvia Berkó, Erzsébet Csányi, Ágnes Dombi, Gábor Pozsgai, Kata Bölcskei, Lajos Botz, Ödön Wagner, Erika Pintér

**Affiliations:** 1 Department of Inorganic and Analytical Chemistry, Faculty of Chemical Technology and Biotechnology, Budapest University of Technology and Economics, Műegyetem rkp. 3, H-1111 Budapest, Hungary; 2 Department of Pharmacology and Pharmacotherapy, Medical School, University of Pécs, Szigeti u. 12, H-7624 Pécs, Hungary; 3 Molecular Pharmacology Research Group, Szentágothai Research Centre, University of Pécs, Ifjúság ú. 20, H-7624 Pécs, Hungary; 4 Institute of Pharmaceutical Technology and Regulatory Affairs, Faculty of Pharmacy, University of Szeged, Eötvös str. 6, H-6720 Szeged, Hungary; 5 Department of Pharmaceutics and Central Clinical Pharmacy, Faculty of Pharmacy, University of Pécs, Honvéd u. 3., H-7624 Pécs, Hungary

**Keywords:** transdermal therapeutic system, capsaicin, silicone, addition polymer

## Abstract

Transdermal therapeutic systems (TTSs) enable convenient dosing in drug therapy. Modified silicone-polymer-based patches are well-controlled and cost-effective matrix diffusion systems. In the present study, we investigated the substance release properties, skin penetration, and analgesic effect of this type of TTS loaded with low-dose capsaicin. Release properties were measured in Franz diffusion cell and continuous flow-through cell approaches. Capsaicin was detected with HPLC-UV and UV spectrophotometry. Raman spectroscopy was conducted on human skin samples exposed to the TTS. A surgical incision or carrageenan injection was performed on one hind paw of male Wistar rats. TTSs were applied to the epilated dorsal skin. Patches were kept on the animals for 6 h. The thermal hyperalgesia and mechanical pain threshold of the hind paws were detected. Patches exhibited controlled, zero-order kinetic capsaicin release. According to the Raman mapping, capsaicin penetrated into the epidermis and dermis of human skin, where the target receptors are expressed. The thermal pain threshold drop of the operated rat paws was reversed by capsaicin treatment compared to that of animals treated with control patches. It was concluded that our modified silicone-polymer-based capsaicin-containing TTS is suitable for the relief of traumatic and inflammatory pain.

## 1. Introduction

Transdermal therapeutic systems (TTSs) provide an excellent mode for convenient, accurate, safe, and painless dosing of drugs [1,2]. Transdermal absorption systems can be categorized according to either their structure or their chemical composition. Regarding structure, they can be adhesive-polymer-dispersion-based, membrane-controlled, polymer-matrix-controlled diffusion-type, and “micro-reservoir”-type systems. Based on their compositions, there are hydrophilic organic copolymers (e.g., polyols, polyethers, etc.) and silicone-based systems (hydrophobic or modified amphiphilic).

**Figure 1:**
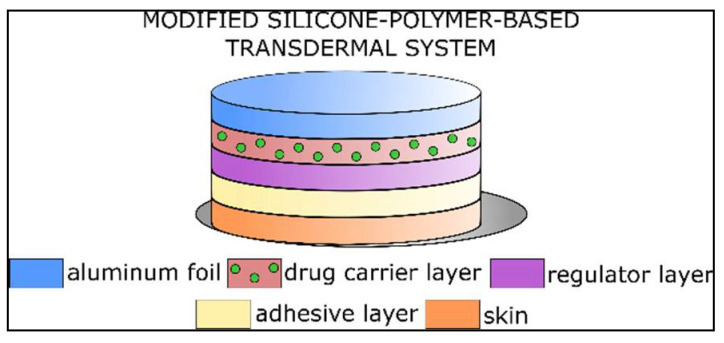
Modified silicone-polymer-based matrix-controlled diffusion TTS.

The drug release of membrane-controlled systems possesses the most favorable characteristics. Their disadvantage is that the drug is in a liquid phase under the control membrane. The TTS cannot be cut, and the dose rate cannot be changed. Adhesive polymer dispersion systems can be sliced without damaging the TTS, but they exhibit suboptimal release kinetics. “Micro-reservoir”-type systems bear both advantageous characteristics, but their production is expensive. This type of TTS is thicker than the others, and the release is controlled by diffusion through the polymer matrix [3]. Construction of the polymer matrix from organic polymers is complicated due to solubility and other chemical issues. Modified silicone-polymer-based techniques provide well-controlled and cost-effective matrix diffusion systems. In the present study, we develop and investigate capsaicin-containing modified silicone-polymer-based transdermal patches (Figure 1).

Such a TTS consists of several layers [4]. The drug is embedded in a polymer layer covered by a regulator layer with special diffusion properties. A controlled drug release profile can be achieved by precisely adjusting the concentration gradient based on different diffusion constants in the two layers (Figure 1).

**Figure 2:**
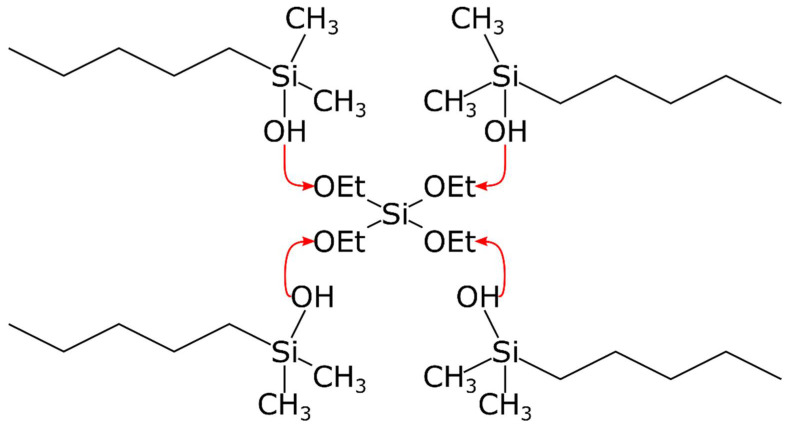
Scheme of silicone polymerization by condensation.

**Figure 3:**
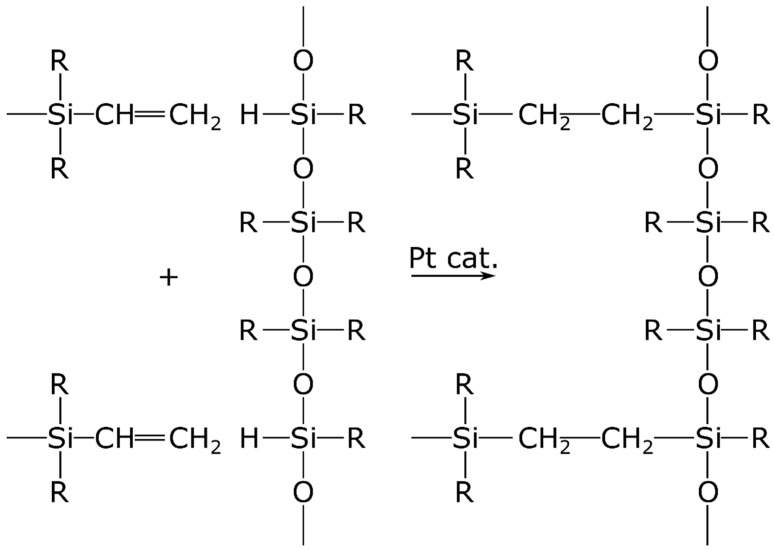
Scheme of silicone polymerization by addition. The “+” sign indicates addition of the two ethenyl groups in the top and bottom left of the figure to respective silicon atoms of the polysiloxane to the right of the “+” sign. The product of the reaction is shown in the right.

The basis of silicone polymers is dimethylpolysiloxane (PDMS), a linear organosilicon polymer crosslinked to form elastic silicone rubber. PDMS can be used as a support matrix. There are two main conventional methods to crosslink PDMS: condensation and addition. In polymers produced through condensation, Si-O-Si bonds are the crosslinkers (Figure 2). In addition polymers, Si-C-C-Si bonds provide the crosslinks between PDMS chains (Figure 3). It is difficult to purify condensation polymers to medical grade. Another problem is that polycondensation requires a tin (Sn) catalyst that is not biocompatible.

**Figure 4:**
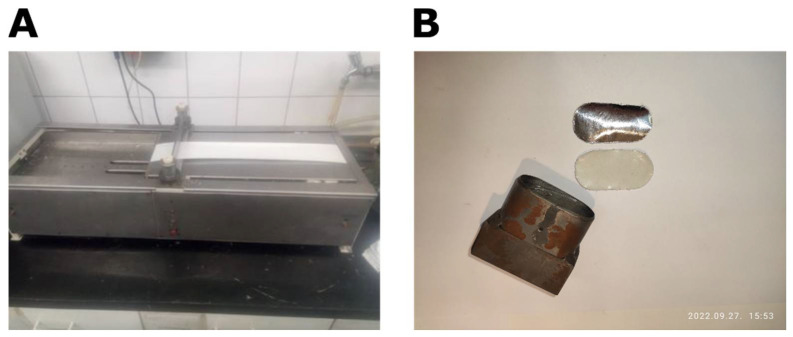
Apparatus for single-layer spreading of the polymer (**A**). Two pieces of the modified silicone polymer TTS (**B**). On the top is the aluminum film that is included to improve mechanical properties. On the bottom is the adhesive layer that touches the skin. The punching tool used to cut the TTS to size is also visible.

The crosslinked structure of addition polymers is more suitable for medical use (Figure 3). It is possible to transform the final matrix structure with additives. This can improve the admixing of the active ingredient to the matrix. Addition silicone polymers can be prepared from two main components: linear PDMS with vinyl groups and a crosslinker containing a hydro-silane compound and a platinum. These substances are biocompatible. The active ingredient and adjuvants are incorporated into the polymer [5]. The viscous mixture cures in 30–60 min at 70× depending on the composition. A carrier layer is required due to poor mechanical properties of the silicone polymer. Usually, a metal or polymer film is utilized (Figure 1). The polymer mixture can be applied to the carrier layer using a single-layer spreading method (Figure 4).

Capsaicin-containing transdermal patches are commercially available. Topical capsaicinoid therapy effectively alleviates pain in several diseases, including rheumatoid arthritis, osteoarthritis, low back pain, and neuropathic pain [6,7,8,9,10]. It also increases blood flow in soft tissues before sports activity to achieve a warming effect. Capsaicin-containing creams and ointments have the disadvantages of contaminating hands and potentially irritating mucous membranes and eyes. This is especially problematic in individuals wearing contact lenses.

Topical non-steroidal anti-inflammatory drugs (NSAIDs) are most commonly applied to relieve osteoarthritis-related pain [11]. The topical use of low-dose capsaicin combined with NSAIDs is a widely accepted therapy in osteoarthritic pain, but the exact mechanism is still under investigation [12]. Ercan et al. presented evidence for the potentiating effect of capsaicin with diclofenac. In a carrageenan-induced inflammation model, topical application of a capsaicinoid-containing patch enhanced the anti-inflammatory effect of diclofenac in rats [13].

The beneficial effect of capsaicin relies on the activation of transient receptor potential vanilloid 1 (TRPV1) ion channels on peptidergic nociceptor nerve endings and the subsequent release of neuropeptides. The antinociceptive effect of TRPV1 activation is transmitted by somatostatin release from the nerve endings [14,15]. Our previous study established that topical capsaicinoid (nonivamide) therapy diminished chronic low back pain in patients. Nonivamide proved to be efficient in functional tests, such as the ODI (effect of pain on everyday life) and VAS (visual analog scale of pain sensation) [16]. Nonivamide treatment induced a three-fold increase in the plasma somatostatin level of the patients.

In the present work, we measure the in vitro release and transdermal penetration of capsaicin using a Franz diffusion cell and Raman microscopy. The antinociceptive effect of the TTS was also tested with the help of in vivo animal studies. Thermal hyperalgesia was measured in response to surgical incision of the hind paw in rats. Carrageenan-induced mechanical allodynia was detected with dynamic plantar esthesiometry in rats.

## 2. Results

### 2.1. In Vitro Experiments

#### 2.1.1. Drug Release and Permeation Investigated with Franz Diffusion Cell

**Figure 5:**
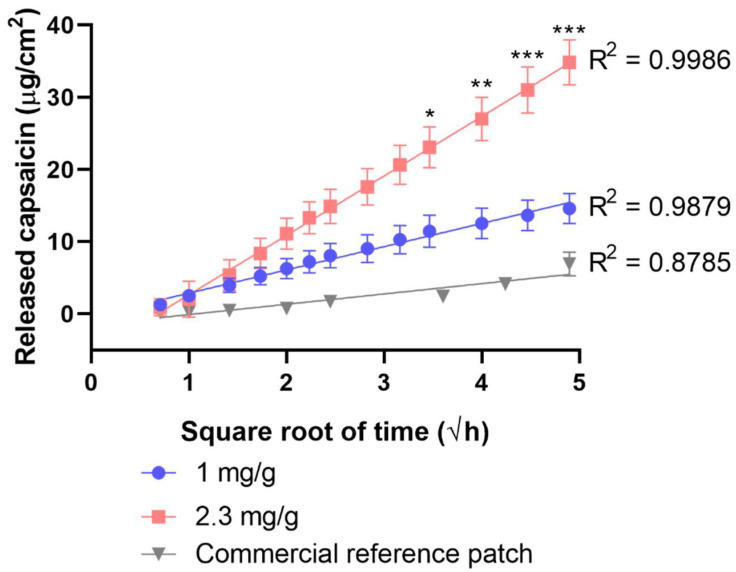
The cumulative amount of released capsaicin in μg/cm^2^ over 24 h (*p* < 0.05, *; *p* < 0.01, **; and *p* < 0.001, ***).

**Figure 6:**
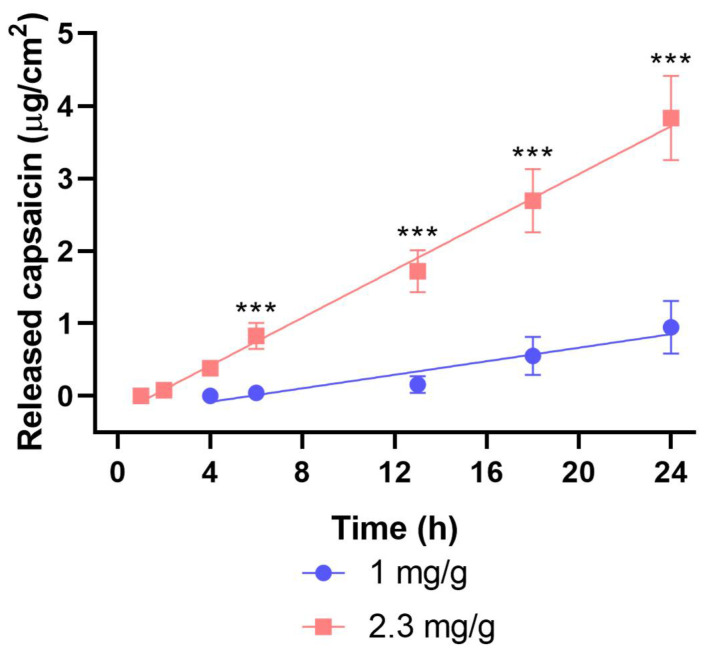
The cumulative amount of permeated capsaicin in μg/cm^2^ over 24 h (*p* < 0.001, ***).

Dermal patches with two different capsaicin contents (1 and 2.3 mg/g) were studied through IVRT (in vitro release test; Figure 5) and IVPT (in vitro penetration test; Figure 6).

In the IVRT measurement, a substantially larger amount of capsaicin was released from the 2.3 mg/g patches within 24 h compared to the 1 mg/g ones. In the IVPT measurement, significantly less of the drug was delivered to the receptor chamber compared to IVRT. This mainly was due to the barrier function of the stratum corneum layer of the skin. The difference between the formulations with two distinct capsaicin contents was similar to that seen in IVRT experiments. Patches with a higher capsaicin concentration yielded higher permeability values. The extent of release (IVRT) itself did not provide relevant information on permeation. It was important to examine the permeation through the skin (IVPT) to reveal the interactions of the drug and the drug delivery system with the skin.

The release and permeation profiles were characterized by flux values (Table 1). Flux values showed the rates of release and permeation of capsaicin from different patches.

We compared the in vitro release kinetics of our modified silicone-polymer patch to a commercially available reference patch (Figure 5). The commercial patch was of the adhesive polymer dispersion type. Even the smaller dose of our TTS (1 mg/g) exhibited a larger capsaicin release than the reference patch. The release kinetics of the modified silicone-polymer patch were closer to zero-order than those of the commercial one. This was suggested by the R-squared values of linear curve fit (Figure 5; 0.8785 for the commercial reference patch, 0.9879 for the 1 mg/g patch, and 0.9986 for the 2.3 mg/g modified silicone-polymer patch).

#### 2.1.2. Results of Drug Release with Flow-Through Cell

**Figure 7:**
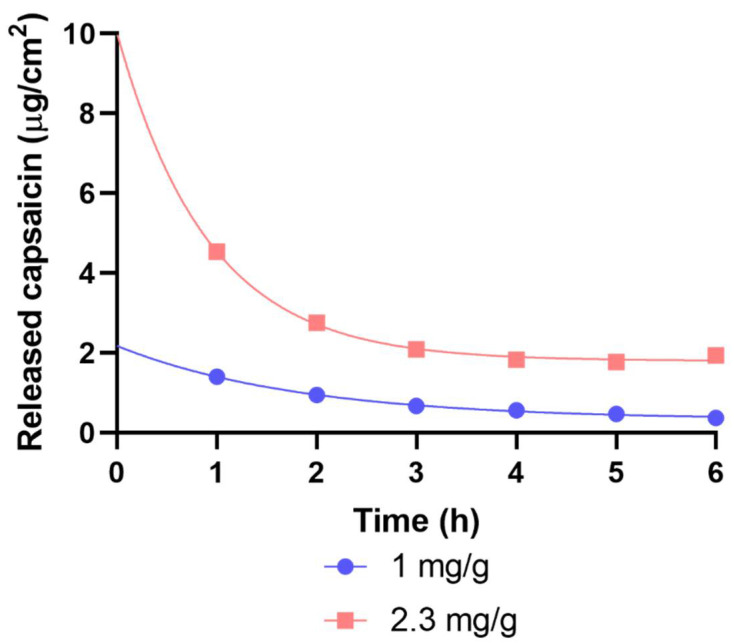
The released capsaicin in µg/cm^2^ over 6 h.

Dermal patches with two different capsaicin contents (1 and 2.3 mg/g) were studied by modified IVRT. A substantially larger amount of capsaicin was released from the 2.3 mg/g patches within 6 h compared to the 1 mg/g ones. The regulation of drug release of the patches was monitored in a flow-through cell. The patch containing more capsaicin had better-regulated drug release over time (Figure 7).

#### 2.1.3. Results of Raman Spectroscopy

During the Raman experiments, the differences in the localization in the skin regions of capsaicin were determined.

The Raman correlation map showed the presence of the penetrated drug in the different layers of the human skin, from the skin surface to the dermis, after the treatment with patches. Spectral maps were constructed in order to detect the presence of capsaicin in the different regions of the human skin. The fingerprint area of the capsaicin spectrum was compared with the spectra of patch-treated and untreated human skin.

**Figure 8:**
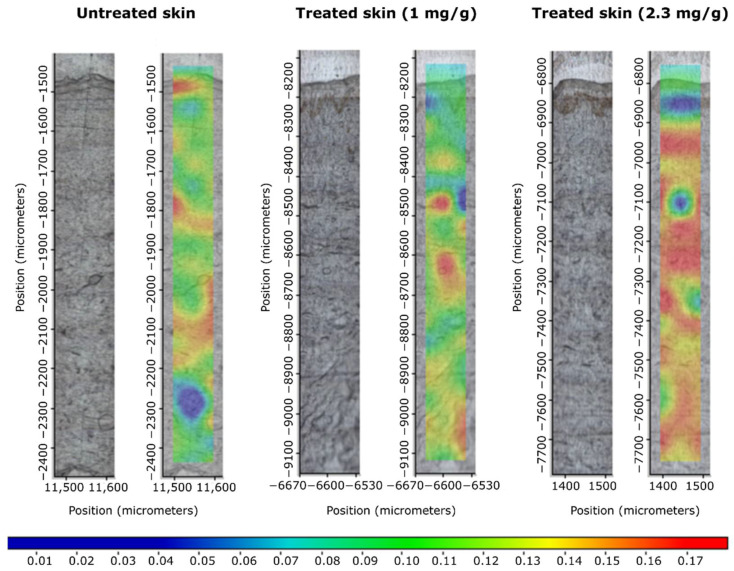
Raman correlation maps for the distribution of capsaicin in human skin after treatment with patches. Untreated skin is also displayed as control. Color coding of drug content: red > yellow > green > blue.

The Raman correlation maps of the patches are shown in Figure 8. The Raman correlation maps demonstrated the presence of capsaicin in different regions of human skin. In correlation with the IVRT and IVPT results, more effective penetration was seen with the 2.3 mg/g patch than with the 1 mg/g one. Capsaicin was detected in the dermis and epidermis.

### 2.2. Results of In Vivo Experiments

#### 2.2.1. The Capsaicin-Containing Dermal Patch Alleviates Heat Hyperalgesia

**Figure 9:**
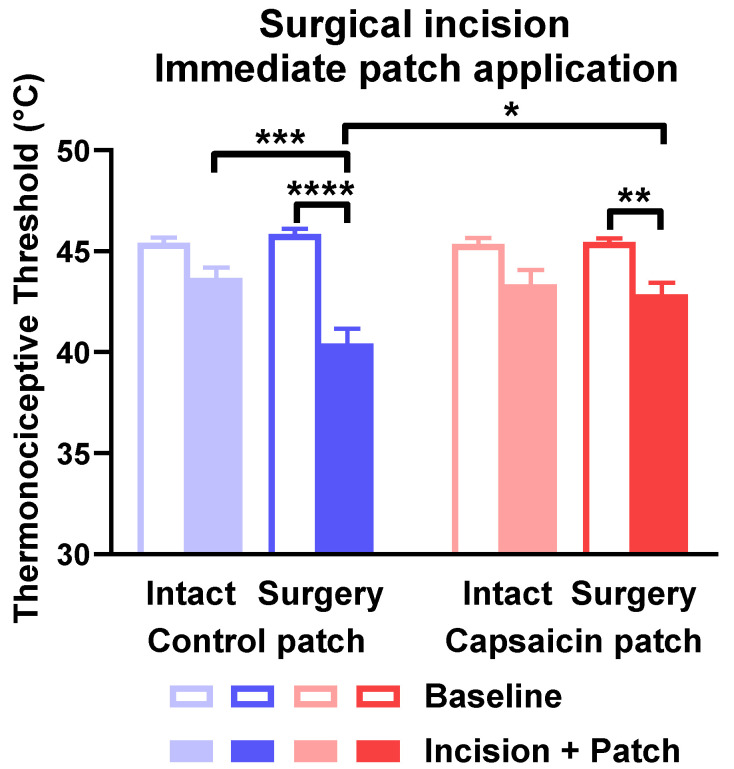
Capsaicin-releasing dermal patch applied right after surgical incision of the hind paw relieved thermal hyperalgesia of the paw. Thermal hyperalgesia is indicated by the painful heat threshold and is shown in degrees of Celsius. * is *p* < 0.05; ** is *p* < 0.01; *** is *p* < 0.001; **** is *p* < 0.0001. *n* = 9–10.

In experiments with patch application immediately after the incision of hind paws, surgical intervention decreased the thermal pain threshold in animals treated with control patches compared to the baseline value, as well as to the contralateral intact paw. The thermal pain threshold of the operated paws in capsaicin-treated rats was still lower than the respective baseline value, but it was significantly larger than the threshold of the operated legs of bandage-treated control animals. The thermal sensitivity of the intact paws of capsaicin-treated rats did not differ from the baseline value taken before surgery. Adhesive bandages had no effect on the thermal pain threshold of hind paws (Figure 9).

**Figure 10:**
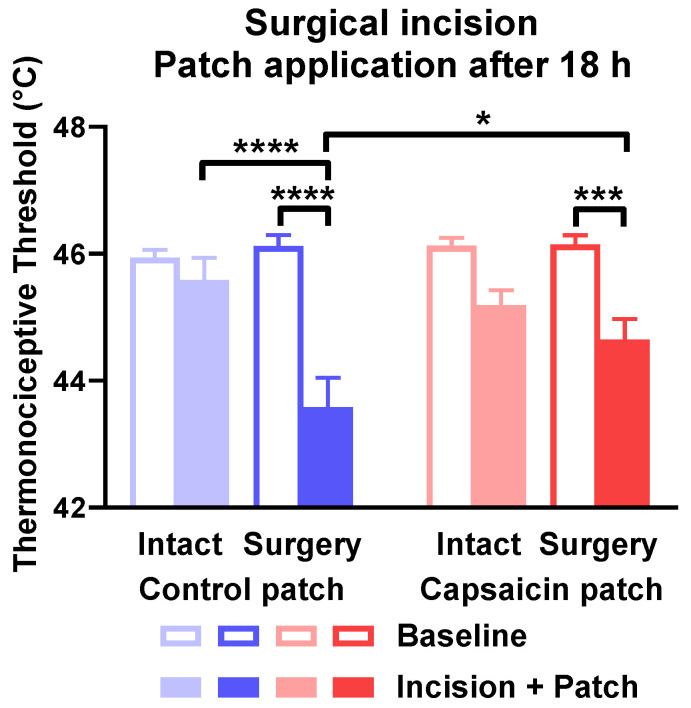
Capsaicin-releasing dermal patch applied 18 h after surgical incision of the hind paw relieved thermal hyperalgesia of the paw. Thermal hyperalgesia is indicated by the painful heat threshold and is shown in degrees of Celsius. * is *p* < 0.05; *** is *p* < 0.001; **** is *p* < 0.0001. *n* = 9.

In experiments with delayed patch application, the surgical incision of paws significantly reduced the pain threshold compared to contralateral intact paws and respective baseline values. Control patches without capsaicin failed to improve this condition. Capsaicin-releasing patches elevated the thermal pain threshold compared to the control patch. The mitigated threshold was still lower than the baseline of the paw. Neither control nor capsaicin-containing patches changed the hyperalgesia of intact paws (Figure 10).

#### 2.2.2. The Capsaicin-Containing Dermal Patch Mitigates Carrageenan-Evoked Mechanical Hyperalgesia

**Figure 11:**
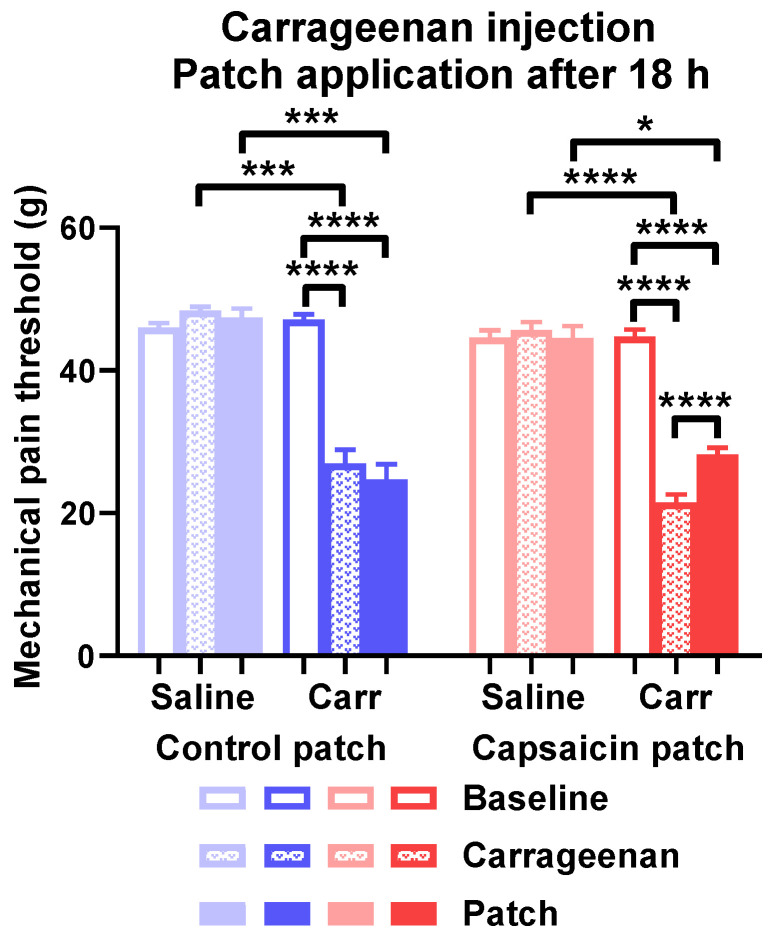
Capsaicin-releasing transdermal patch applied 18 h after challenge alleviated carrageenan-induced mechanical paw hyperalgesia. Mechanical pain threshold of the hind paws is shown in g. * is *p* < 0.05; *** is *p* < 0.001; **** is *p* < 0.0001. *n* = 9–14.

Carrageenan reduced the mechanical pain threshold detected 18 h after challenge in rats treated with both adhesive tape and capsaicin-containing patches compared to the contralateral paw. The mechanical thresholds of carrageenan-treated paws were still reduced compared to contralateral paws after 6 h of treatment with capsaicin patches or the control. The threshold of carrageenan-injected paws was significantly elevated by capsaicin treatment compared to the value detected before patch application. Contralateral paws injected with saline did not show any sensitization (Figure 11).

## 3. Discussion

In this study, controlled-release capsaicin-containing patches were prepared and tested. Our transdermal patch was a modified silicone-polymer-based diffusion-gradient-controlled system, providing optimal drug release and cost-effective therapy. Patches were produced with an addition-crosslinked silicon polymer method containing two different capsaicin concentrations and tested under “in vitro” and “in vivo” conditions [17].

Addition-type silicone has very apolar properties. Since capsaicin shows a polar character, polar environments had to be created in the apolar matrix to promote its delivery in the right amount and to move properly inside the silicone matrix [18]. Capsaicin is very soluble in alcohols, but shorter-chain monohydric alcohols are volatile compounds and exert detrimental effects in human skin. The simplest trivalent alcohol, glycerol, was used as a solvent. This skin-friendly compound dissolves capsaicin relatively well. However, glycerol is insoluble with silicone oligomers; therefore, we used an emulsifier to achieve even distribution in the matrix. Since glycerol saturated with capsaicin does not contain enough capsaicin to adequately deliver the desired amount of the drug, solid capsaicin was dispersed in the matrix. A small, required amount of highly potent solid capsaicin would have made the homogeneous distribution uncertain. Capsaicin was subjected to powder dilution (trituration) with calcium carbonate to ensure precise dosing and homogeneous drug distribution. A drug-free control layer, which also contained glycerol, was applied to the drug-containing layer so that capsaicin could diffuse through it unimpeded, but only in the desired amount. One mg/g and 2.3 mg/g capsaicin-containing patches were prepared and tested.

In the flow-through cell dissolution test, it was found that the drug was dissolved from the patch with the higher drug content at a much higher rate, and the dissolution rate returned to a uniform 2 mg/cm^2^ after 3 h. The initial high drug release was due to the diffusion saturation of the regulatory layer with capsaicin from the underlying drug-containing layer. In the first hour of the dissolution test, the drug dissolved from this saturated layer. The rate of dissolution was high because of the short diffusion path of the drug. The drug released from the control layer was replaced by the layer below, but due to the increase in the diffusion distance, the drug release value reduced and remained constant.

In the case of the patch with lower drug content, the control layer could not saturate with capsaicin. Drug release started from a lower level, and this value decreased slightly but continuously over time because of the increasing diffusion pathway.

In consequence, the phenomena observed in the dissolution test were due to the fact that, in the case of the patch containing a larger drug concentration, the control layer was almost saturated with the drug and continuously replenished the capsaicin flowing into the liquid medium due to diffusion from the drug-containing layer. In the case of the patch with a smaller drug content, this replacement did not occur, and the increasing diffusion distance could not be compensated for by the diffusion rate. On the other hand, it can be stated that the control layer fulfilled its expected function in both patches. Instead of an exponential decrease in the matrix diffusion, only a slowly decreasing release curve could be observed.

A similar trend was shown in the drug release studies (IVRT) in the Franz cell, with significantly lower drug release from the lower-capsaicin patch. According to the human dermal permeation study (IPVT), the permeation of the drug was more superficial from the lower-drug patch, and the penetration of capsaicin also required longer time. In patches with a higher ingredient content, the compound already passed through the skin in half the time compared to the amount of the transferred active ingredient and was much larger than proportionately expected.

The results showed that, in the case of the layer combination we used, the higher the drug content, the better the desired zero-order drug release kinetics. Prolonged, controlled release was also confirmed by Raman microscopic examinations. In the case of the patch with a higher capsaicin content, the drug penetrated evenly and permanently into the skin tissue. The devices and methods used to make the patches modeled industrial production. Therefore, the patches could be adapted for small-scale production with minor modifications.

Dermal patches containing large concentrations (8%) of capsaicin are licensed for the treatment of neuropathic pain, such as postherpetic neuralgia and diabetic neuropathy [19].

The mechanism of action of these patches involved a strong and sustained activation of the transient receptor potential vanilloid 1 (TRPV1) ion channels of primary nociceptor nerve endings. The opening of channels causing pathologically increased intracellular Ca^2+^ concentration of nerve endings leads to cytoskeletal and mitochondrial damage. Nociceptor nerve endings are defunctionalized for 12–14 weeks, providing long-lasting analgesia.

Thus, the nociceptor function of the nerve fibers and substance P (SP) release—which is thought to be important signal for pain neurotransmission—also becomes impaired for extended period [20,21]. These processes have been considered as potential mechanisms of the analgesic action of topical capsaicin treatment, but several clinical studies proved that SP receptor antagonists failed to be analgesics [22]. Anand and Bley (2011) suggested that capsaicin has limited potential for transdermal delivery across human skin and that it causes defunctionalization only of the cutaneous nociceptors [23].

Data corroborate that transdermal systems containing much smaller than defunctionalizing doses of capsaicin might also be effective against neuropathic pain. A dermal patch containing only 0.04% capsaicin alleviated postherpetic neuropathic pain in 60.1% of the patients, 28.2% of whom exhibited increasing analgesia throughout 12 weeks [24]. A mixed patient group suffering from either postherpetic or diabetic neuropathic pain experienced analgesic effects of a transdermal patch containing 0.625% capsaicin [25].

It might be puzzling how such a small capsaicin content might exert effective analgesia. Antinociceptive effects developed in the deeper musculoskeletal and joint areas could not be explained by the desensitization of the cutaneous afferents [23]. The activation of TRPV1 ion channels and consequent elevation of intracellular Ca^2+^ concentration induces neuropeptide release but does not damage the nerve endings. Many of these peptides contribute to vasodilatation and plasma extravasation (e.g., substance P and calcitonin gene-related peptide). Other peptides, such as somatostatin or endogenous opioids might possess analgesic and anti-inflammatory actions [26]. The systemic antinociceptive effect of somatostatin was proved in animal studies [14]. Human data also proposed that the systemic analgesic effect of topical capsaicinoid treatment is related to the remarkable increase in somatostatin concentration in the plasma [16].

In addition to the peripheral action, somatostatin exerts a central analgesic effect as well [27]. Somatostatin immunoreactive structures were detected in lamina II of the lumbar spinal cord of a rat and were proposed as the anatomical basis for somatostatin-induced analgesia [28]. The expression of SSTR4 receptor mRNA was detected at various levels of the murine and human neuronal pathways of pain sensation [29]. The analgesic effect of somatostatin, including neuropathic pain, could be reproduced by selective sst_4_ receptor agonists [29].

**Figure 12:**
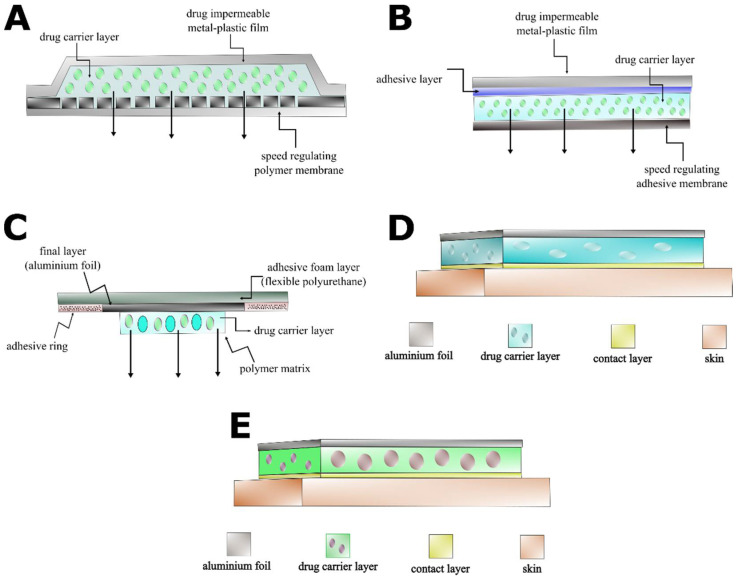
Schematic structures of the most frequent types of transdermal drug delivery systems. The figure shows the membrane-controlled (**A**), drug in adhesive (**B**), micro-reservoir (**C**), classical matrix diffusion (**D**) and modified silicone-polymer (**E**) systems.

Figure 12 and Table 2 outline the benefits of the modified silicone-polymer TTS over other types of transdermal systems. Membrane-controlled systems exhibit excellent drug release kinetics, but they cannot be dosed by cutting the TTS (Figure 12A, Table 2). Drugs in adhesive-type patches can be dosed by cutting and have simple structures, resulting in cost-effective production. However, the drug release of this TTS type is unregulated (Figure 12B, Table 2). Micro-reservoir systems possess better release kinetics and can be cut to size. On the other hand, they are thicker than other TTS types. This results in inferior flexibility and worse fit to the skin surface (Figure 12C, Table 2). Classical matrix diffusion transdermal patches can be dosed by cutting, as well as display good flexibility and fit to the skin. Nevertheless, their production might be complicated and expensive, and their drug release kinetics fall short of those of membrane-controlled systems (Figure 12D, Table 2). The modified silicone-polymer TTS described in our study combined the advantageous flexibility and cuttable property of matrix diffusion systems with a simpler, less expensive production process and close to zero-order drug release kinetics rivaling those of membrane-controlled patches (Figure 12E, Table 2). Altogether, the modified silicone-polymer TTS had optimal characteristics for accurate and low-cost transdermal drug delivery.

In summary, the transdermal patch described in the present paper offered the opportunity of low-dose topical capsaicin treatment without contaminating the hands or clothing and allowing for precise dosing by cutting the patches to size. Moreover, our technology offered outstanding release kinetics that might be exploited with other pharmacons. Our transdermal system was subjected to both routine—Franz cell and Raman spectroscopy—and innovative—flow-through cell—in vitro testing. In our opinion, circumstances in the flow-through cell modeled those in the cutaneous tissues during the release of the active substance from the dermal patch more precisely. We chose well-established animal models of nociception to investigate our transdermal system. The increasing-temperature water bath is not a widely known method, despite being validated in a surgical paw incision pain model with opioids and NSAIDs [30]. The exposition of the whole paw surface to heat is a profound advantage in the surgical incision model because different areas of the paw might exhibit different grades of hyperalgesia. This practically makes mechanical testing at least challenging, if not impossible. To the contrary, classical carrageenan-induced paw inflammation enables easy and effective testing of mechanical hyperalgesia by dynamic plantar esthesiometry [31]. Animals undergoing carrageenan-induced paw inflammation do not exhibit heat hyperalgesia. These methods might be well-suited for the testing of other transdermal therapeutic systems.

## 4. Materials and Methods

### 4.1. Chemicals

RT-601 A™ addition-crosslinkable polydimethylsiloxane- (α, ω) -divinyl and RT-601 B™ crosslinker were from Wacker GmbH, Munich Germany. Glycerol was purchased from Molar Chemicals Ltd., Halásztelek, Hungary. Polysorbate 20 was obtained from Molar Chemicals Ltd., Halásztelek, Hungary. Capsaicin was purchased from Chillies Export House Limited, Virudhunagar, Tamil Nadu, India.

### 4.2. Production of Capsaicin-Containing Transdermal Patches

TTS samples used in animal experiments were prepared on a paper substrate laminated on aluminum foil of 0.4 mm thickness. Capsaicin was mixed into silicone matrix carriers. Our raw material was RT-601 A™ addition-crosslinkable polydimethylsiloxane- (α, ω)–divinyl. Capsaicin was dissolved in glycerol by heating and was added to the silicone stock. Crystalline capsaicin was also added to our mixture. Capsaicin was diluted with calcium carbonate. Calcium carbonate as an inert excipient was added to the samples only to ensure accurate balancing. If needed, liquid glycerol and polysorbate 20 were added. RT-601 B crosslinker was added to the mixture under stirring. After the components were weighed, mixtures were homogenized and spread on a supporting film at a thickness of 0.4 mm. The layer was crosslinked at 70 °C. The procedure was finished in 60 min. After that, we spread a second regulator layer (Figure 1) that did not contain capsaicin, only glycerol and polysorbate. The second layer was crosslinked at 70 °C for 60 min. Samples were rested for 48 h and examined afterwards. We made two compositions: a lower (1 mg/g capsaicin) and a higher (2.3 mg /g capsaicin) capsaicin-containing sample. The compositions of samples of 1 mg/g and 2.3 mg/g were as follows (Table 3 and Table 4).

### 4.3. Measurement of the In Vitro Release of Capsaicin-Containing Transdermal Patches

**Figure 13:**
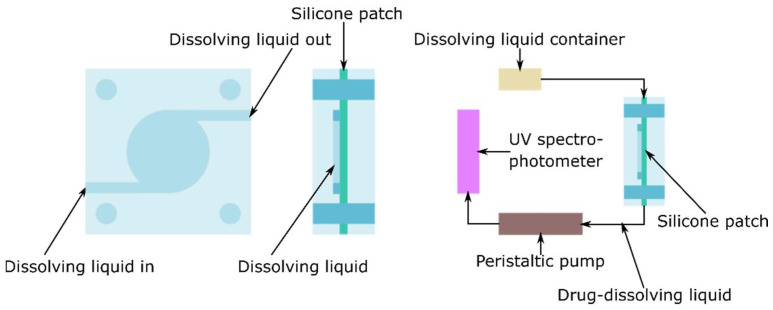
Flow-through cell.

In vitro testing was performed in two ways. First, we measured in a Franz cell [32], which modeled the static and vertical subcutaneous drug dissolution. In the second method, patches were examined in a flow-through cellular device that mimicked the dissolute drug concentration in the blood (Figure 13).

#### 4.3.1. Investigation of Drug Release and Permeation with Franz Diffusion Cell

In vitro release tests (IVRTs) and in vitro permeation tests (IVPTs) were performed. A vertical Franz-type diffusion cell (Hanson Microette TM Topical and Transdermal Diffusion Cell System, Hanson Research Corporation, Los Angeles, CA, USA) was used to model the capsaicin release from the patches in the case of the IVRT, as well as drug permeation through human heat-separated epidermis (HSE) in the case of the IVPT. The preparation of HSE was the following: excised human subcutaneous fat-free skin was placed in a water bath (60 ± 0.5 °C), and the epidermis was separated from the dermis.

Around 250 mg of each patch (1.77 cm^2^) was used as the donor phase. The patches were placed in the donor chamber directly in the case of the IVRT. In the case of the IVPT, the donor and receptor phases were separated by HSE.

The receptor phase was thermostated phosphate buffer (PBS pH 7.4 ± 0.15) and 25% *w*/*w* 96% ethanol at 32 °C ± 0.5 °C. The investigation lasted for 24 h. The stirring speed was 450 rpm. The concentration of the drug was examined by high-performance liquid chromatography (HPLC). HPLC analysis was carried out with a Shimadzu NEXERA X2 HPLC system (Shimadzu Corporation, Tokyo, Japan). A Kinetex C18 150 mm × 4.6 mm packed with a 3 µm (Phenomenex Inc., Torrance, CA, USA) column was used. Acetonitrile in a ratio of 30:70 with a flow rate of 1 mL/min was applied during isocratic elution with HPLC-grade water. Prior to the elution, the eluent was degassed and filtered through a 0.45 µm pore size glass filter funnel. The run time was 4 min, and the retention time of capsaicin was 2.3 min. Detection was performed via absorption at 280 ± 4 nm. A sample volume of 20 µL was injected, and the elution was carried out at a sample temperature of 25 °C and a column temperature of 45 °C.

#### 4.3.2. Mathematical Evaluation

The data were the averages of the results of 6 experiments ± standard deviations. Release and permeation profiles of the patches were obtained. The cumulative amounts of capsaicin released and permeated per cm^2^ at 24 h were calculated. The flux (J) was the slope of the cumulative amounts of released and permeated capsaicin (µg/cm^2^) versus the square root of time (h^1/2^) in the case of the IVRT and versus time (h) in the case of the IVPT [33].

#### 4.3.3. Flow-Through Cell

Samples (12.56 cm^2^ each) of patches were tested for modified IVRT in a flow-through cell (4 cm diameter and 5 cm^3^ sample volume) at 37 °C. The flow rate (PBS, 5% *w*/*w* glycerol) was 25 mL/h, and the capsaicin content was determined hourly with a spectrophotometer (Perkin-Elmer Lambda 25, PerkinElmer Inc., Waltham, MA, USA). Detection was performed via absorption at 227 nm.

### 4.4. Investigation of Skin Permeation with Raman Microscopy

Excised human subcutaneous fat-free skin (epidermis and dermis) was obtained from a Caucasian female patient who underwent abdominal plastic surgery. Samples of patches (1.77 cm^2^) were placed on the skin surface for 3 h at 32 °C. The treated skin samples were frozen and sectioned (10 μm thick cross-sections) with a Leica CM1950 cryostat (Leica Biosystems GmbH, Wetzlar, Germany).

The microtomed skin samples were placed on an aluminum surface with the SC towards the top of the plate.

Raman spectroscopic measurements were performed with a Thermo Fisher DXR Dispersive Raman Spectrometer (Thermo Fisher Scientific Inc., Waltham, MA, USA) equipped with a CCD camera and a diode laser.

A laser light source of 780 nm wavelength was used with a maximum power of 24 mW, minimizing the effect of fluorescence. The microscopic lens used for the measurements had 50 × magnification, and the aperture of the pinhole was 25 μm. In the case of chemical mapping, a 200 μm × 1800 μm area was investigated; the step size was 50 μm both vertically and horizontally. Each spectrum was produced from 16 scans with an exposure time of 2 s. Altogether, 205 spectra were registered. For analyzing the treated vs. untreated skin samples, capsaicin was used as a reference. Data acquisition and analysis were accomplished using OMNICTM8.2 Dispersive Raman software package (Thermo Fisher Scientific Inc., Waltham, MA, USA).

### 4.5. In Vitro Measurement

#### 4.5.1. Animals

Male Wistar rats of 125–150 g in weight were purchased from ToxiCoop Zrt., Budapest, Hungary. The rats were kept at the Department of Pharmacology and Pharmacotherapy, Medical School University of Pécs, under standard pathogen-free conditions with freely available food pellets and water. Experiments conformed to the 40/2013., II. 14. Hungarian Government regulation on the protection of animals used for scientific purposes, to the European Communities Council Directive 2010/63/EU, and to the requirements of the International Association for the Study of Pain (IASP). Experiments were approved by the Ethics Committee on Animal Research of the University of Pécs and the National Scientific Ethical Committee on Animal Experimentation of Hungary (license number BA02/2000-8/2018, 28/02/2018). The dorsal skin of the animals was epilated with commercial epilation cream from the nape to the hip under ketamine and xylazine anesthesia (80 and 10 mg/kg i.p.) 2 days before the animals participated in experiments.

#### 4.5.2. Surgical Incision of the Hind Paw

Animals were anesthetized with sodium pentobarbital (50 mg/kg i.p.), and the plantar surface of one hind paw was treated with povidone iodine. Sides subjected to surgery were randomized. The paw was incised at a wound length of 10 mm with a scalpel. The depth of the incision reached the muscle layer. The wound was closed with two 6.0 sutures and treated with povidone iodine [26,30]. Capsaicin-releasing dermal patches were applied according to 2 schedules. In one set of experiments, patches were attached to the dorsal skin right after the paw incision when the pentobarbital anesthesia still lasted (immediate application). In other experiments, patches were applied 18 h after the incision (delayed application). The size of the patches was 3 cm × 6 cm. Patches were fixed to the animals with commercial adhesive bandages. Adhesive bandages without dermal patches were used as the control. Patches were kept on the animals for 6 h in both experimental designs. After the six-hour interval, the thermal pain threshold of the hind paws was measured with increasing-temperature water bath (Experimetria Kft., Budapest, Hungary). Both hind legs of rats were submerged into water separately.

The water was heated from 30 °C to 51 °C with a velocity of 24 °C/min. The heating was stopped by a foot switch when the animal removed the paw from the water, and water temperature was recorded. The animals were habituated to handling by the experimenter and the instrument 3 times, and baseline values were taken.

#### 4.5.3. Carrageenan-Induced Paw Inflammation

Carrageenan was dissolved in physiological saline under gentle heating (3% *m*/*v*). Carrageenan was injected intraplantarly into one hind paw of rats. The contralateral paw was injected with saline [31]. Capsaicin-loaded dermal patches were applied to the backs of animals 18 h after carrageenan injection. The size of the patches was 3 cm × 6 cm. Patches were fixed to the animals with commercial adhesive tape. Bandages without dermal patches were used as the control. Six hours later, the mechanical pain threshold of the hind paws was detected by dynamic plantar esthesiometry (Ugo Basile, Gemonio, Italy). Rats were placed into the compartments of the instrument 10 min before the test. The force exerted by the stimulator reached 50 g in 5 s. The value inducing nocifensive behavior was automatically displayed. Baseline measurements were performed 3 times before the actual experiment. Lowered pain threshold was confirmed by the detection of mechanical hyperalgesia before the application of transdermal patches.

#### 4.5.4. Statistical Analysis

The results were evaluated and analyzed statistically with one-way (in vivo studies) or two-way (in vitro experiments) analyses of variance, followed by Bonferroni’s multiple comparisons test using Prism from Windows software (GraphPad Software Inc., La Jolla, CA, USA, access date: 1st of January, 2022). The data were the means of the results of 6 experiments ± standard deviations (*p* < 0.05 was *, *p* < 0.01 was **, and *p* < 0.001 was *** vs. control) [34,35].

## 5. Conclusions

Our silicone-based TTS displayed long-lasting, controlled, dose-dependent release and permeation of capsaicin. The higher-dose (2.3 mg/g) capsaicin-containing TTS was capable to deliver the active ingredient to the target receptors in the dermis and exerted systemic antinociceptive action.

We presumed that activation of the TRPV1 ion channels on the sensory nerve endings in the patch-treated dorsal skin exerted the release of inflammatory neuropeptides, such as SP and CGRP, inducing local warmth and painful redness. In addition, antinociceptive mediators, such as somatostatin and opioid peptides, were released from the central peripheral endings of the primary afferents, regulating the pain pathway. The systemic analgesic effect of the low-dose capsaicin patch could be explained by these sensocrine regulatory mechanisms.

Further experiments involving a TTS loaded with various detergents and other excipients possessing unexplored potentials may offer further optimization of substance release and increased therapeutic value.

## Figures and Tables

**Table 1:** Flux values of released and permeated capsaicin.

Dose	IVRT ^1^	IVPT ^2^
1 mg/g patch	3.2215	0.0466
2.3 mg/g patch	8.233	0.1672

^1^ IVRT—in vitro release test; ^2^ IVPT—in vitro permeation test.

**Table 2:** Comparison of properties of the most frequent types of transdermal therapeutic systems.

Type of TTS	Drug Release Kinetics	Dosing by Cutting	Flexibility
Membrane-controlled	close to zero-order	no	good
Drug in adhesive	unregulated	yes	good
Micro-reservoir	regulated	no	poor
Classical matrix diffusion	regulated	yes	good
Modified silicone-polymer	close to zero-order	yes	good

**Table 3:** Chemical composition of the low-dose capsaicin patch (1 mg/g).

Patch Layer	Layer Thickness	Component	Content
Drug carrier layer	0.4 mm	Capsaicin (solid, triturated)	3.60%
		Capsaicin (solution)	3.75%
		Glycerol	13.16%
		Polysorbate 20	3.95%
		RT 601 A	69.32%
		RT 601 B	7.83%
Regulator layer	0.1 mm	Glycerol	12.05%
		Polysorbate 20	4.00%
		RT 601 A	74.21%
		RT 601 B	9.69%
**Total capsaicin content**			1 mg/g patch

**Table 4:** High-dose capsaicin patch.

Patch Layer	Layer Thickness	Component	Content
Drug carrier layer	0.4 mm	Capsaicin (solid, triturated)	8.36%
		Capsaicin (solution)	5.58%
		Glycerol	10.52%
		Polysorbate 20	5.69%
		RT 601 A	62.89%
		RT 601 B	6.98%
Regulator layer	0.1 mm	Glycerol	11.88%
		Polysorbate 20	3.43%
		RT 601 A	76.21%
		RT 601 B	8.46%
**Total capsaicin content**			2.3 mg/g patch

## Data Availability

Data are contained within the article.
